# The use of remote presence for health care delivery in a northern Inuit community: a feasibility study

**DOI:** 10.3402/ijch.v72i0.21112

**Published:** 2013-08-05

**Authors:** Ivar Mendez, Michael Jong, Debra Keays-White, Gail Turner

**Affiliations:** 1 Remote Medicine Program, Division of Neurosurgery, Dalhousie University and Queen Elizabeth II Health Sciences Centre, Halifax, NS, Canada; 2 Faculty of Medicine, Memorial University, St. John's, NL, Canada; 3 Health Canada, First Nations and Inuit Health Branch Atlantic, Halifax, NS, Canada;; 4 Nunatsiavut Department of Health and Social Development, St. John's, NL, Canada

**Keywords:** Aboriginal health, air transport, health care costs, patient care, patient satisfaction, remote presence, robots, telemedicine

## Abstract

**Objective:**

To evaluate the feasibility of remote presence for improving the health of residents in a remote northern Inuit community.

**Study design:**

A pilot study assessed patient's, nurse's and physician's satisfaction with and the use of the remote presence technology aiding delivery of health care to a remote community. A preliminary cost analysis of this technology was also performed.

**Methods:**

This study deployed a remote presence RP-7 robot to the isolated Inuit community of Nain, Newfoundland and Labrador for 15 months. The RP-7 is wirelessly controlled by a laptop computer equipped with audiovisual capability and a joystick to maneuver the robot in real time to aid in the assessing and care of patients from a distant location. Qualitative data on physician's, patient's, caregiver's and staff's satisfaction were collected as well as information on its use and characteristics and the number of air transports required to the referral center and associated costs.

**Results:**

A total of 252 remote presence sessions occurred during the study period, with 89% of the sessions involving direct patient assessment or monitoring. Air transport was required in only 40% of the cases that would have been otherwise transported normally. Patients and their caregivers, nurses and physicians all expressed a high level of satisfaction with the remote presence technology and deemed it beneficial for improved patient care, workloads and job satisfaction.

**Conclusions:**

These results show the feasibility of deploying a remote presence robot in a distant northern community and a high degree of satisfaction with the technology. Remote presence in the Canadian North has potential for delivering a cost-effective health care solution to underserviced communities reducing the need for the transport of patients and caregivers to distant referral centers.

Profound disparities in the provision of health care services to Canadians who live in the north have had a major negative impact on their life expectancy. Indigenous inhabitants such as the Inuit are the most vulnerable ethnic group in the Canadian North and have an average life expectancy that is 11 years lower than the rest of Canadians (1). A low population density within a vast and remote territorial expanse in the context of jurisdictional issues, socio-economical and historical realities have contributed to this inequality in health status. Inuit have higher rates of preterm birth, stillbirth and infant mortality than other Canadians (2). The incidence of chronic diseases, substance abuse and injuries continue to increase in Inuit communities (3). Sixty-eight percent of Inuit children aged 6–14 years report being hungry on a regular basis (4) compared to 1.2% of children in other Canadian families (5). Infectious diseases such as lower respiratory tract infections are 11 times more frequent in Nunavik than in other Canadian regions (6) and Inuit women have higher rates of human papilloma virus infections compared to other Canadian women (7).

Health expenditure per capita in the Canadian North is higher than in the rest of Canada (8). For example, the per capita health expenditure in Nunavut is 2.7 times greater than the national average and constitutes more than 30% of its GDP, the highest in the world. These high levels of health care expenditure do not correlate with improved health indicators. This failure to achieve better health outcomes despite high levels of spending is multifactorial and complex (9). Although historical, cultural, socio-economic and geographical dynamics are at play, the model of health care delivery to remote communities in the Canadian North may be a critical factor in this discrepancy.

Remote northern Canadian communities typically have community health clinics that are staffed by advanced care regional nurses who practice in an extended role with the support of off-site family physicians located in regional health centers that help with the delivery of elective and emergent care. Although there are periodic visits from physicians to the remote clinics, the system relies heavily on air transport as the main conduit for accessing physician care in the regional or specialized referral centers (9). The reliability and sustainability of air transport in providing services to remote locations is heavily dependent on financial resources and climatic conditions which are unpredictable in the North. Telemedicine has opened the door for the development of solutions that may help address the unmet needs of remote communities. Although telemedicine applications in the Canadian North were initially conducted in the late 1970s and early 1980s (10,11) with some focused programs having been successful over the years (12–14), the adoption of telemedicine as a widespread and effective pathway for health care delivery to remote northern communities has not occurred. Several barriers and challenges have impeded the adoption of telemedicine as a routine strategy for the effective and timely delivery of health care to underserviced remote regions (15,16). As part of our telehealth partnership program, we have explored the feasibility of using a remote presence robot to deliver real-time physician expertise to Nain, a remote Inuit community in northern Newfoundland and Labrador, Canada.

## Methods

### The Nain community health clinic

The community of Nain is located at the northern tip of the province of Newfoundland and Labrador, on Canada's east coast and is the administrative capital of Nunatsiavut, the newest Inuit land claim. Nunatsiavut is located on a geographically diverse area of approximately 72,000 km^2^ of land and 48,690 km^2^ of sea that is home to a population of about 2,200 Inuit. The community of Nain has 1,188 residents (17) and is served by a community health clinic staffed by 6 regional nurses.

The Nain clinic provides primary care to the community (it is not an inpatient facility) with 4 holding beds and a treatment room with basic resuscitation equipment. The referral center for the Nain Clinic is at the Labrador Health Center in Happy Valley-Goose Bay, 367 km south of Nain. Emergency or urgent care patients are evacuated to the referral center by air. Although a physician from the Labrador-Grenfell Regional Health Center visits Nain once a month, patients requiring further diagnostic tests or assessment are transported by air to Happy Valley-Goose Bay or the tertiary care referral center in St. John's, 1,207 km away.

Physician support for the Nain nurses has been mainly through telephone complemented in the past 5 years with videoconferencing equipment and a telehealth coordinator stationed in Happy Valley-Goose Bay. Air transport from Nain to the referral centers is dependent upon weather conditions and restricted to the day time as the Nain runway has no night time capabilities.

### Remote presence robot

To provide physician remote presence expertise to the Nain clinic, we used the RP-7 remote presence robot (In Touch Health Inc., Santa Barbara, CA, USA). The RP-7 has been designated by the US Food and Drug Administration (FDA) as a class II medical device and fulfills the US FDA requirements for active patient monitoring in clinical situations in which immediate clinical action may be required (18). The RP-7 was flown to Nain and deployed in the community clinic, the robot was nicknamed “Rosie” by the clinical staff in Nain. Physicians in the referral center in Happy Valley-Goose Bay and nurses in the Nain clinic were trained on the use of the system before its operation.

**Fig. 1:**
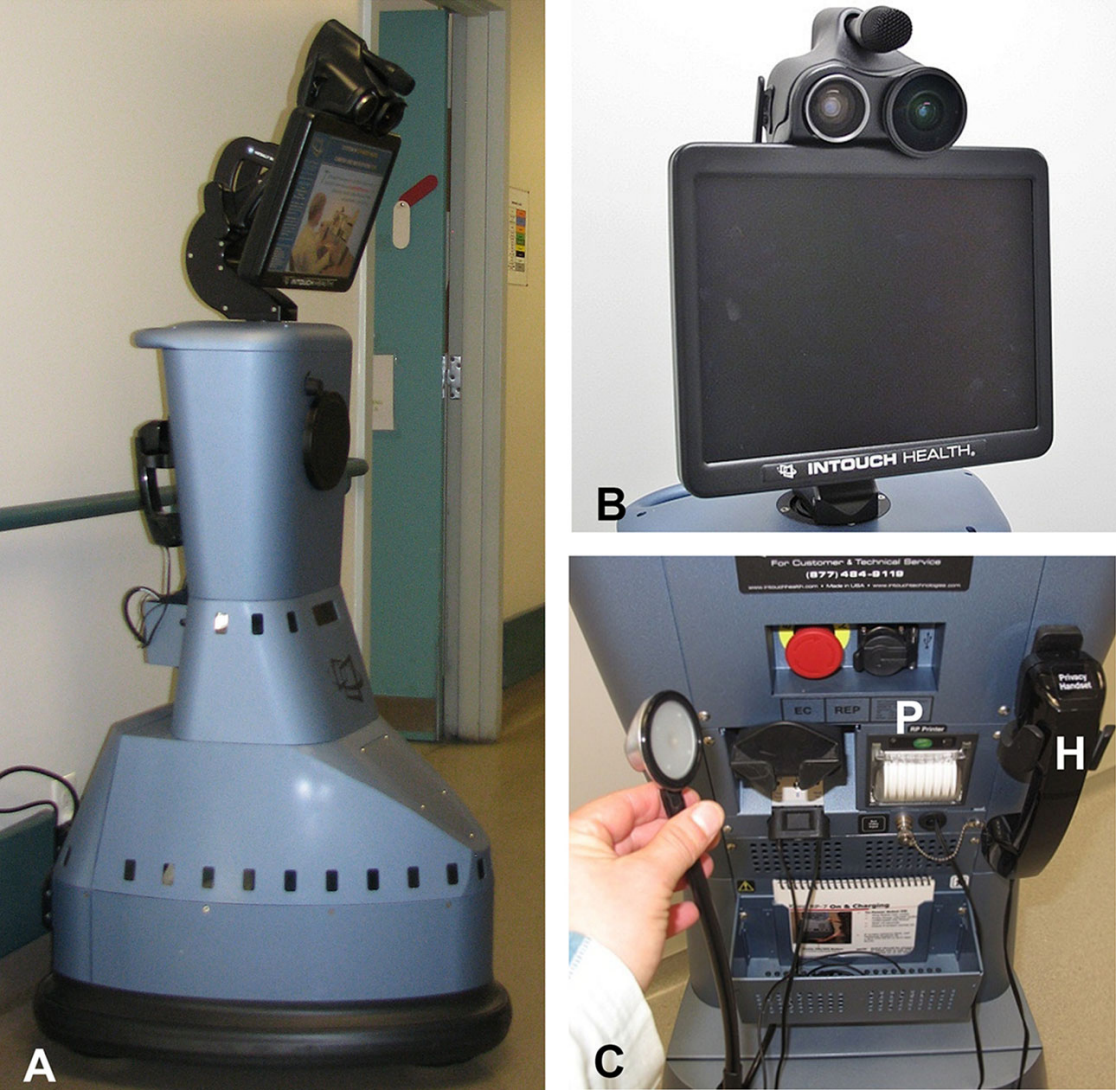
Photographs of the (A) RP-7 remote presence robot that is 165 cm in height and has a 63×76 cm wheeled triangular base; (B) a close-up view of the RP-7 monitor and the two affixed high-resolution cameras; and (C) is capable of connecting diagnostic peripherals, such as a stethoscope. The RP-7 robot has a printer (P) for printing orders and prescriptions from the referring physician and a telephone handset (H) for private communication with the distant physician.

The RP-7 is controlled wirelessly by a laptop computer (control station) equipped with headphones, microphones and a joystick to maneuver the robot in real time. The RP-7 is 165 cm in height and has a wheeled triangular base of 63×76 cm, roughly comparable to the size of a human. The robot can travel at speeds of about 3 km/h and has an 8-h rechargeable battery (Fig. 1).

The head of the RP-7 has a mobile flat screen monitor that displays the image of the operator and a picture-in-picture window that displays the image of the person standing in front of the robot. The head of the robot is movable and is fitted with two sophisticated digital cameras as well as audio, microphone and amplification components allowing for real-time two-way audiovisual communication (Fig. 1B). The robot also has a digital stethoscope, privacy handset and a printer capable of providing hard copies of orders and recommendations with the digital signature of the physician conducting the remote presence clinical session (Fig. 1C). Connectivity between the control station and the RP-7 robot is provided by a standard 802.11 Wi-Fi internet link.

**Fig. 2:**
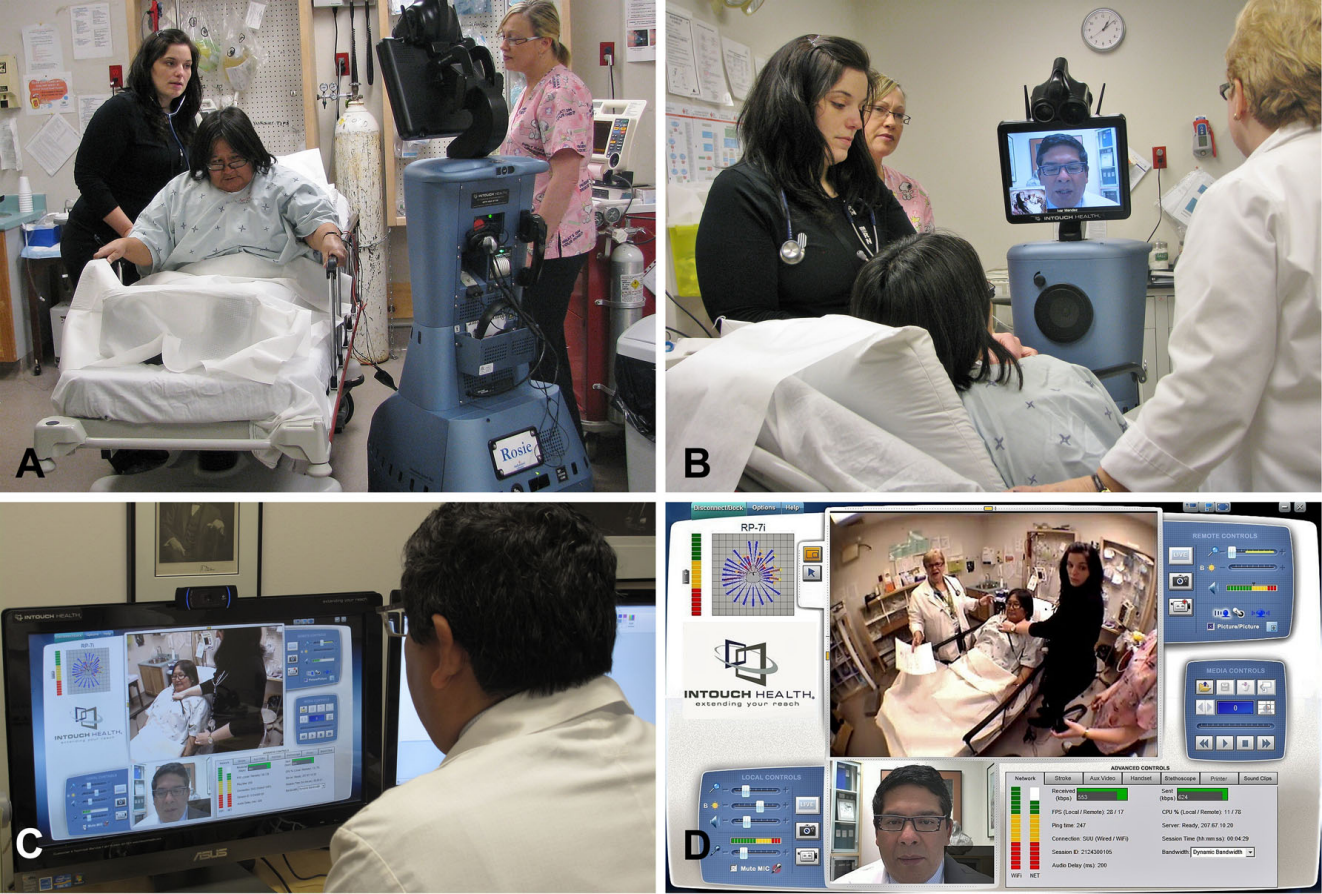
Photographs of the RP-7 remote presence robot, nicknamed “Rosie” (A) at a patient's bedside assessment; (B) the remote physician interacting with the patient and the clinical team in Nain; (C) control station used by the remote physician conducting the clinical assessment in the Nain clinic and (D) snapshot of the control screen for the RP-7 remote presence robot.

The control station allows the clinician to have real-time control of the robot's movement, videoconferencing systems, digital stethoscope and printer. The clinician operating the robot is able to telestrate using a cursor that is seen in the robot's head screen. Telestration is important, as the clinician can use it to impart real-time visual instructions in the remote location environment such as pointing to the patient's anatomy while conducting a physical examination with the aid of a nurse (Fig. 2). The control station is also capable of storing video and still images of the remote presence sessions for further analysis and archival purposes.

### Assessments

The project duration was 15 months, from January 1, 2010 to March 31, 2011, and data were collected using a combination of surveys, qualitative interviews and compiling information on travel and communication costs as well as the network time used during the RP-7 sessions. As regular videoconferencing continued to be used in Nain, comparison data were collected for the same period of time.

Evaluation forms were completed after each remote presence session by the physician, the Nain nurse and the patient or caregiver accompanying the patient. The remote session surveys provided data on the demographics of the patients, diagnosis, outcome after the session, ease of RP-7 use as well as the participant's satisfaction with the session.

A third-party evaluator conducted qualitative interviews at the end of the project with physicians and nurses participating in the study. Data on the number and cost of air transport, accommodation and meals for Nain patients and caregivers accompanying the patient for the period of this study were collected. The number and duration of RP-7 activations were recorded. Activations were divided into 3 session categories; clinical sessions, education sessions and technical/maintenance activations.

## Results

### Remote presence usage

There were 252 activations of the RP-7 robot during the 15-month study period of which the majority (89%) were for patient care (Table I). There was an average of 11.8 robotic remote presence clinical sessions per month. During this time, regular videoconference clinical sessions remained relatively stable from 9.3 per month prior to commencement of the project to 7.8 per month during the course of the study. The combination of the RP-7 clinical sessions and regular videoconferencing sessions represented a 150% increase in telehealth access to the residents of Nain. Forty-nine percent of the clinical sessions were for mental health care assessments and follow-up. Although only 3% of RP-7 activations were for purely education purposes, the Nain nurses placed a high value on these sessions, and reported that in 50% of the clinical sessions they had learned something new.

**Table I:** Purpose for activation of the remote RP-7 robot during a 15-month period in Nain, Labrador

Type of session	% of sessions (252 total)
Initial consult	20
Follow-up	44
Emergent/urgent	25
Charting/nurse consult	7
Education/in-service	3
Technical/maintenance	1
Total	100

### Impact on air transport

Air transport of patients from remote communities to the referral center is a major economic burden to health care delivery in remote northern Labrador communities. There are two methods for transporting patients by air from Nain to the referral center in Happy Valley-Goose Bay; (1) Medical Evacuation (Medivac) which is a dedicated flight for patient transport or (2) a regularly scheduled commercial flight. The costs associated with these two flights are significantly different. A regularly scheduled flight costs $875 CND round trip, in comparison to a Medivac flight, which costs $1,800 CND one-way (not included escort costs and call-back costs for ambulance dispatch staff). Other costs that are incurred when patients are referred for outpatient assessments and diagnostic tests to the Happy Valley-Goose Bay Center are accommodations ($150 CND per night) and meals ($29 CND per day). These costs are also applicable to the patient's caregiver/translator that usually accompanies the patient on these trips. The flight costs are subsidized by the provincial government and the associated costs of travel are born by the Nunatsiavut government-administered Non-Insured Health Benefits Program.

Analysis of air transportation parameters for 47 remote presence sessions where data were complete indicated that in 60% of those cases air transport to the referral center was avoided, where it normally would have been considered (Table II). In those cases, the physician conducting the remote session felt satisfied that the patient could be effectively managed and followed in Nain and did not require transport to the referral center. Furthermore, 7 of the 14 patients who were assessed using the RP-7 robot for emergency or urgent reasons and would have potentially required Medivac transport, were effectively managed in Nain Of these 7 patients, 6 patients (43%) were deferred to a regularly scheduled commercial flight and only 1 patient (7%) required a Medivac flight (Table II).

**Table II:** Remote presence assessments performed and the outcome of the sessions

Assessment	Medivac	Scheduled flight	Number of transport required	Total
Initial consult	0	0	10	10
Follow-up	9	3	11	23
Emergent/urgent	1	6	7	14
Total	10	9	28	47
% of total	21	19	60	100

### Satisfaction assessments

#### Physicians

The learning curve for driving the RP-7 robot is not steep as the controls and operability are very intuitive. Participating physicians required approximately 1 h of training prior to being comfortable in operating the RP-7 robot. All physicians participating in the project indicated that the technical capabilities of the RP-7 robot were superior to that of the conventional videoconference setup for telehealth delivery routinely used in Nain (Fig. 2). There was a high degree of satisfaction with use of the RP-7 robot for making real-time clinical decisions, patient follow-up and enhanced interaction with both nurses and patients. Furthermore, 100% of physicians felt the RP-7 improved clinical collaboration with nurses, facilitated workflow and decreased stress levels when making diagnostic and management decisions from a distant central location.

#### Nurses

Deployment of the RP-7 robot in Nain had a very positive impact on the nurses with 100% of the nurses feeling that the RP-7 robot was superior to the conventional videoconference telehealth setup. Eighty-four percent of the nurses felt that remote presence facilitated the diagnosis and management of the patient and 80% of nurses reported that the RP-7 robot facilitated physician–patient interaction. All nurses reported that improved access to physician support in real time as provided by the RP-7 robot could facilitate retention and recruitment of nurses to remote northern communities in Canada.

#### Patients

There was a high degree of satisfaction amongst patients being evaluated in the Nain clinic using the RP-7 robot by physicians located in the referral center. Ninety-five percent of the patients indicated that they would use the RP-7 robot again for their clinical evaluations, with 84% of patients reporting that they were “very comfortable” in their interaction with the assessing physician using the RP-7 robot. In 53% of the remote presence sessions, an interpreter or family member accompanied the patient. Ninety percent of those caregivers felt that the use of the RP-7 robot was very helpful in promoting interaction with the physician conducting the session.

## Discussion

### The Nain experience

This is the first experience using a remote presence robot to provide real-time physician expertise to a remote community in the Canadian North. The transport, deployment and operation of the RP-7 robot in the Nain clinic were straightforward and well-received. The learning curve for physicians and nurses in the routine operation of the RP-7 robot was not steep and could be accomplished with about 1 h of training. Physicians and nurses reported that the intuitive controls of the robot, its mobility, stable connectivity, high-resolution cameras and two-way audiovisual capabilities were distinct advantages over the conventional videoconference setup for telehealth routinely used in Nain. The ability to drive the RP-7 robot to the patients’ location and have high-resolution real-time audiovisual connectivity with the patient, nurses and family members improved both the physicians’ and nurses’ comfort with the clinical assessment as well as enhanced the interaction with the patient. All physicians and nurses reported that use of the RP-7 robot improved workflow and reduced stress levels.

Nurses reported that the higher level of collaboration with physicians during remote presence sessions was paramount to gaining community confidence in the new technology. This collaboration was particularly effective during mental health sessions that represented 49% of all remote presence sessions. Those sessions were reported to be “extremely helpful” in managing the high prevalence of mental health issues in the community. The nurses also felt that remote presence technology may have a crucial impact in retention and recruitment of nurses to isolated northern communities and empower them to provide a wider range of services in collaboration with real-time input from physicians using remote presence.

The acceptance of the RP-7 robot by patients was very high with 95% of the patients indicating that they would use the RP-7 robot again and 84% reporting being “very comfortable” interacting with their physician via the robot. Having access to physician expertise in their own community without the need for transport to the referral center has profound implications on the attitudes of the community for seeking medical attention. These attitudes have been influenced by historical events in northern Canadian communities related to tuberculosis outbreaks as far back as two generations ago. It is possible that the availability of remote presence systems such as the RP-7 robot and an increase in their sophistication for point-of-care diagnosis, such as real-time blood chemistry analysis, portable imaging systems, electrophysiological assessment tools and other diagnostic implements will not only change these attitudes but also remove barriers of distance and time for providing effective health care to underserviced populations such as Nain.

Although health expenditures per capita in the Canadian North are high, they are not correlated with improved health care access or health indicators. The model of care in the Canadian North relies heavily upon transportation of patients by air to regional referral centers for hospital admissions, outpatient clinical assessments, follow-up and diagnostic tests. The costs of flights and other associated expenses such as meals and accommodation for the patients and in many cases their companions can be overwhelming (9). Providing effective clinical care using remote presence devices and avoiding unnecessary air transportation to referral centers can significantly improve the cost-effectiveness of health care provision in the Canadian North. Our results in Nain provide some initial evidence that remote presence can dramatically reduce the number of flights to a distant referral center by about 60%. Although a reduction in the number of flights could lead to obvious savings in the costs associated with transportation and accommodation of patients, redirection of this money to the timely diagnosis and improved management of patients in remote communities is the foremost benefit of remote presence.

### Robotic telepresence

Developments in robotic and telecommunications technology may help address the provision of medical expertise in underserviced remote communities. In fact, there is growing evidence for the benefits of the RP-7 robotic system in clinical applications and telementoring. One of the earliest applications of the RP-7 has been in critical care where there is a chronic shortage of intensivists and increasing demands, resulting in challenges for providing on-site coverage (19). Several studies have demonstrated the utility of remote presence using the RP-7 in providing critical care coverage, resulting in decreased lengths of stay in the intensive care unit (ICU), reduced unexpected events, cost savings and high satisfaction scores by patients, ICU staff and intensivists (20,21). Furthermore, a recent study has showed that robotic telepresence was viewed positively by ICU patients and their families and they felt that the use of the RP-7 was beneficial to their care and indicated their support for its continued use (22). Remote presence has also been recently used for the treatment of stroke through acute thrombolytic therapy, where it has been crucial for facilitating remote neurological assessments reducing the time for onset of therapy, thereby resulting in improved neurological outcomes (23–25). Use of the RP-7 in the perioperative follow-up of patients undergoing laparoscopic gastric bypass showed significant savings by decreasing the length of stay (26).

Robotic telepresence has also been used in surgical mentoring, the RP-7 and its earlier version the RP-6 were used in mentoring laparoscopic surgery for adult and pediatric procedures and considered very useful and reliable for mentoring minimally invasive surgery (27,28). Long-distance telementoring in laparoscopic urological procedures has also been performed with the RP-7 system (29), and we have also used a remote robotic telecollaboration system capable of controlling robotic arm movements for long-distance telementoring of cranial and spine surgeries (30).

Remote presence systems provide an expert the ability to telementor in real-time, a non-expert individual to perform sophisticated diagnostic tests. Complex ultrasound examinations under real-time remote guidance have been conducted aboard the International Space Station where crewmembers in orbit performed thoracic, vascular and echocardiographic examinations under the guidance of an earth-based expert (31). Furthermore, a recent study has shown the feasibility of telementoring paramedics with no previous experience to perform ultrasound trauma assessments with great accuracy under the guidance of a remote expert (32).

Our experience in Nain showed that physicians in a regional referral center were able to provide real-time medical expertise to nurses in a remote Canadian Inuit northern community for the diagnosis and management of patients seen on an emergency or elective basis at the community health clinic. High satisfaction scores by physicians, nurses and patients using the RP-7 robot and a reduction of patient transport to the referral center strongly suggest that remote presence may help provide effective and cost-efficient health care delivery to remote communities lacking on-site physician expertise.

### Potential barriers

Potential barriers for the implementation of remote presence in remote communities would not likely be technological. The exponential advances in the telecommunications, robotics and mobile device industry provide a solid platform for implementing remote presence systems such as the RP-7 robot used in Nain. The barriers are likely to be related to issues pertaining to medical liability, jurisdictional legal considerations, provider remuneration, data and patient confidentiality, competing health priorities, and the lack of regional and national strategies and standards for implementation of this type of telemedicine application. A recent study examining the barriers for implementing robotic telemedicine has determined that the top barriers for adoption of telemedicine solutions in emergency and critical care are regulatory barriers for physician's privileges, financial barriers for billing of remote presence services and resistance to the change of established clinical paradigms (16). However, the explosive increase in the use of consumer mobile devices for medical applications may force streamlining of the regulatory and remuneration issues. Public expectations and pressure for cost-effective and decentralized health care provision may play a significant role in removing cultural barriers to remote presence medicine, especially in underserviced communities such as Nain in the Canadian North. The acceptance of patients and their families to remote presence solutions for health care delivery is quite favorable (22). In this study, 95% of the patients indicated that they would use the RP-7 robot again for their clinical evaluations.

Health expenditure per capita in the Canadian North is higher than in the rest of Canada (8); however, they are not correlated with improved health indicators or health care access within northern communities. Although the costs of emerging technologies such as the RP-7 are initially high (the RP-7 cost is approximately $145,000 USD), it will decrease substantially as the adoption of the technology increases and savings in air transport would foreseeably offset its costs.

### Future directions

This study established the feasibility of using the RP-7 remote presence system to provide real-time access to physician expertise in a remote northern Inuit community. This initial experience was felt to be so positive for the community of Nain that the Nunatsiavut Government has decided to deploy an RP-7 robot in the community permanently. This is a great opportunity to explore long-term impact of remote presence technology for health care delivery in remote northern Canadian communities. Although consumer portable communication devices such as smart phones and tablets are being increasingly used to transmit medical information, it is likely that portable remote presence devices that fulfill standards and regulatory parameters for dedicated medical use will see more widespread use due to their enhanced capabilities. Cellular phone networks have grown exponentially in the world. The latest survey by the International Communication Union indicated that by 2010, 90% of the world's population was covered by mobile cellular networks and that the number of mobile cell subscriptions was approaching 6 billion (33). Mobile broadband is also increasing with 4G connectivity rapidly becoming the norm and will likely continue to increase allowing for the transmission of more complex data. This exponential advance in telecommunications technology may be a powerful and cost-efficient tool in narrowing the gap of inequality in health care delivery to remote northern communities in the near future.
